# How to measure propagation velocity in cardiac tissue: a simulation study

**DOI:** 10.3389/fphys.2014.00267

**Published:** 2014-07-22

**Authors:** Andre C. Linnenbank, Jacques M. T. de Bakker, Ruben Coronel

**Affiliations:** ^1^Department of Experimental Cardiology, Heart Center, Academic Medical CenterAmsterdam, Netherlands; ^2^ICINUtrecht, Netherlands; ^3^Department of Medical Physiology and Cardiology, UMCUUtrecht, Netherlands

**Keywords:** activation mapping, conduction velocity, single vector method, average vector method, simulation, anisotropy

## Abstract

To estimate conduction velocities from activation times in myocardial tissue, the “average vector” method computes all the local activation directions and velocities from local activation times and estimates the fastest and slowest propagation speed from these local values. The “single vector” method uses areas of apparent uniform elliptical spread of activation and chooses a single vector for the estimated longitudinal velocity and one for the transversal. A simulation study was performed to estimate the influence of grid size, anisotropy, and vector angle bin size. The results indicate that the “average vector” method can best be used if the grid- or bin-size is large, although systematic errors occur. The “single vector” method performs better, but requires human intervention for the definition of fiber direction. The average vector method can be automated.

## Introduction

The assessment of the conduction velocity of the cardiac impulse is an important aspect of the study of arrhythmogenesis, especially where it involves arrhythmias based on reentry (Kleber et al., 1986; Janse and Wit, 1989). Slow conduction facilitates reentrant arrhythmias, because it causes a reduction of the wavelength (Mines, 1914). Heterogeneity of conduction velocity and of excitability may provide a substrate for the occurrence of unidirectional block, which is essential for the onset of reentrant activation (Arita and Kiyosue, 1983; Coronel et al., 2009).

Spread of activation in myocardial tissue depends on the myocardial fiber direction (anisotropy). It is fastest in the direction of the myocardial fibers and slow transverse to fiber direction. The normal anisotropic ratio (longitudinal conduction velocity divided by transverse conduction velocity) is for ventricular myocardium about 2:1 (Spach et al., 1981). The difference is caused by the shape of the cardiomyocyte in combination with the preferential localization of connexins at the short sides of the myocytes. As a result of cardiac pathology, the dimensions of the myocyte may change (Wiegerinck et al., 2006), the localization of gap junctions may change toward the lateral sides of the myocytes (lateralization) (Peters et al., 1997) and the extracellular matrix (collagen content) may increase (Weber and Brilla, 1991). Myocardial scarring and fibrosis cause lateral isolation of the myocytes leading to an increase of local anisotropic ratio (Spach et al., 1981; Frazier et al., 1988; de Bakker et al., 1993; Kawara et al., 2001) and of the facilitation of current-to-load mismatch. The latter is important for the generation of unidirectional conduction block (Hoogendijk et al., 2010). In addition, various sodium channel mutations (Remme et al., 2006) and heart diseases (Valdivia et al., 2005) are associated with a decrease in peak sodium current and conduction velocity. An increase in anisotropy is also considered proarrhythmic (Wilders et al., 2000).

For the study of arrhythmia mechanisms the determination of the longitudinal and transverse conduction velocity (CV_l_ and CV_t_), therefore, is essential. Ideally, conduction velocities are calculated from high density surface mapping (either electrically or optically) studies following pacing from the center of the grid of mapping electrodes (Kleber et al., 1986). Under these conditions the activation wave shows an ellipsoid spread of activation starting from the central pacing site. At larger distances from the pacing site the surface activation pattern may show signs of epicardial (or endocardial) breakthrough following intramural propagation, resulting in unrealistically high calculated conduction velocities.

**Figure 1:**
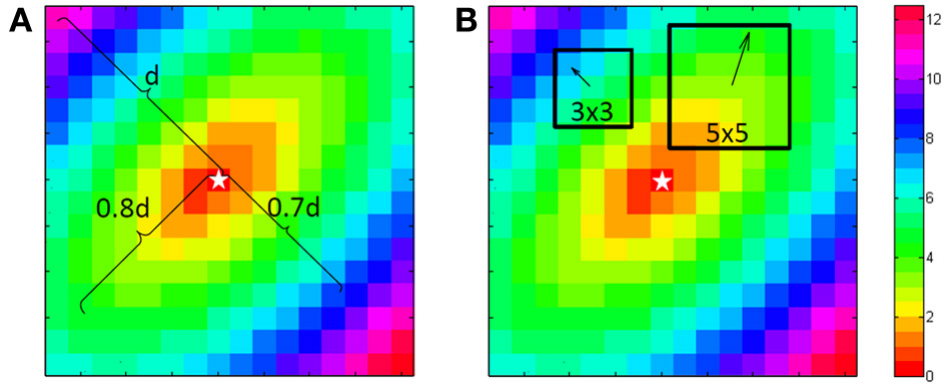
**Computation of the longitudinal and transversal velocities**. For the single vector method a point is chosen at 80% of the length from the central “stimulus” (star shape) to the corner in the direction of the fibers. This is used to define the longitudinal velocity. At right angles at 70% another point is chosen for the transversal velocity. Because this is a simulation both can use the center as the starting point. For the average vector method either in a 3 by 3 or a 5 by 5 environment of a point the principle direction and size is fitted with a least square algorithm. This is done for every point, except the edge points.

**Figure 2:**
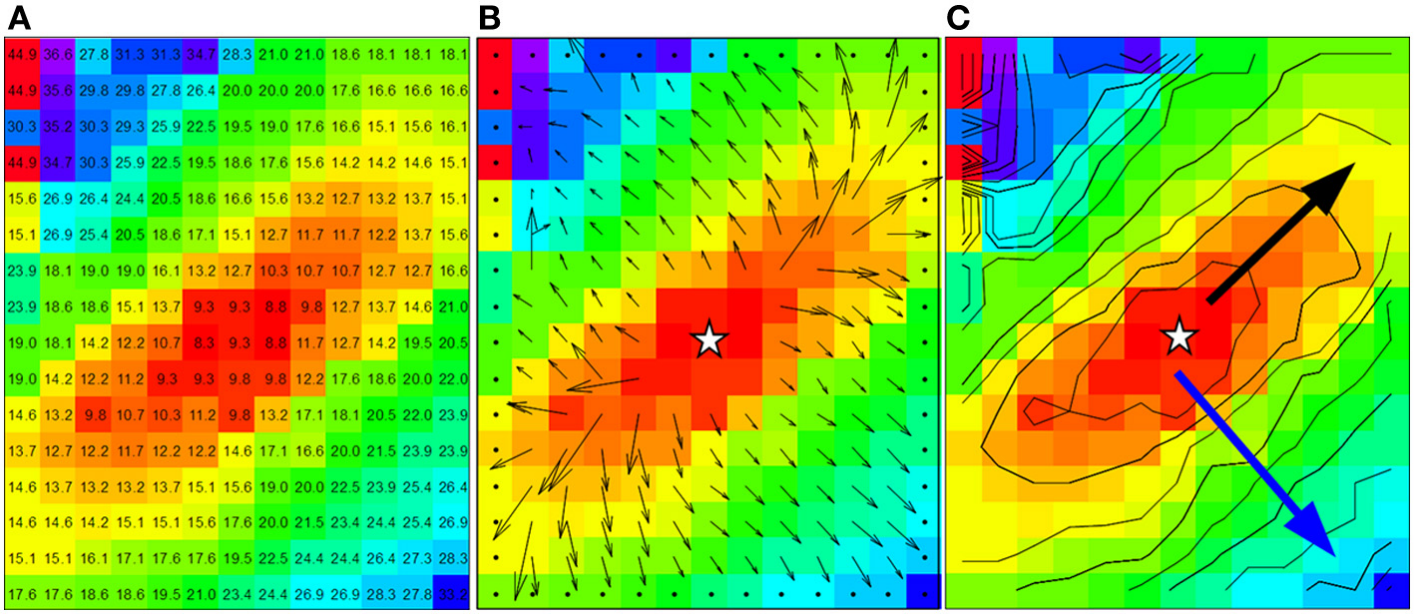
**Data from dog experiment. (A)** Are the measured activation times. **(B)** The vector field derived from the activation times in panel a by using a 3 by 3 neighborhood least square fit. **(C)** Shows the approximately ellipsoid isochrones at 2 ms distance. The black arrow is used for the manually selected longitudinal conduction velocity (2.8 mm in 6 ms, *Vl* = 0.47 m/s) and the blue arrow for the transversal conduction velocity (3.2 mm in 17 ms, *Vt* = 0.19 m/s). Star shape in **(B,C)** indicate the actual stimulus site.

Two methods are commonly used to estimate conduction velocities (Figure 1). The “single vector” method is a more or less manual approach and consists of identifying recording sites on an inner and outer ellipsoid on a line perpendicular to the isochronal lines along the short and long axis of the ellipsoids. The recording sites are selected along a line perpendicular to the isochronal lines (Kleber et al., 1986). CV_l_ and CV_t_ are then calculated from the difference in timing and the known distance between the recording points. Figure 2C shows the isochrones and the longitudinal end transversal vectors as they might be chosen in a manual procedure. Data recorded form a dog heart with a 13 by 16 electrode grid with 0.5 mm distance between the electrodes (Coronel et al., 2010) shown in Figure 2A.

The “average method” divides the whole grid in smaller subgrids (typical 3 × 3 or 5 × 5 recording points). Using the activation times in a subgrid, the best fitting vector (direction and velocity) of the activation front at the central point of the subgrid is estimated. This procedure yields a local activation vector for every point except for border points. (An example of vectors computed from the data in Figure 2A is shown in Figure 2B). These local activation vectors are collected in bins of vectors in the same direction. The average length of the vectors in a bin is used as the velocity in that direction. The average vectors with the largest and smallest conduction velocity value are selected as the longitudinal and transversal velocity in the grid.

Preliminary tests of the average vector method revealed that bins could be empty if the bin-size was too small. In particular in the direction of the fibers. Larger bin sizes on the other hand could result in bias if vectors were included that were not in pure transverse or longitudinal directions. The single vector method depends on selecting just 2 points, which might result in a larger SD. It is not clear which of the two methods performs better for the calculation of CV_l_ and CV_t_. We therefor performed a simulation study to investigate the effect of bin size, grid size, anisotropy, and noise on the accuracy of determination CV_l_ and CV_t_ using both methods. We discuss the pro and cons of the two methods.

## Methods

Conduction velocities determined by using the single vector and average vector method were used to analyze:

1. An activation sequence derived from electrograms recorded with a 13 by 16 multi-electrode-grid from an *in situ* canine heart (Coronel et al., 2010). Stimulation was at BCL 600 ms from the center pair of electrodes.
2. Simulated activation maps derived from activation times for grids of varying size and varying anisotropic ratios following stimulation from a central site. Activation times at the recording sites of the different grids were computed from elliptical isochronal lines with diagonal long and short axis. Grid-sizes from 8 by 8 to 32 by 32 with anisotropic ratios of 1.2, 1.5, 2, and 3 were investigated.

For the “single vector” method a Matlab program was used. It selects a point at approximately 80% of the distance between the stimulation site to the border for the longitudinal direction, because closer to the edge one cannot see if the spread is still homogeneous. The activation in the transversal direction may be influenced by activation via deeper layers, therefore transversal velocity is often estimated from a shorter vector and 70% was used for the transversal velocity. The stimulus point was used as the origin of the vectors (see Figure 1A).

For the average vector method local vectors were computed in a 3 by 3 and a 5 by 5 subgrid surrounding each recording site, excepting one or two rows/columns from the margins of the grid, depending on the subgrid size (see Figure 1B). The computed conduction velocity was plotted as function of the vector angle. The effect of bin-size of the vector angle was investigated by using bin sizes of 15 and 30°. Both seemed a-priori reasonable values as compromise between good angular resolution and enough vectors in the bins. For each combination ⅛, ¼, ½, and 1 ms Gaussian noise was added and 1024 simulations were performed in order to simulate measurement errors. Data were rounded to 0.5 ms to simulate a sampling rate of 2 kHz. In total 1,638,400 (1024 simulations for 25 grid-sizes, 4 anisotropy levels, 4 noise-levels, 2 bin sizes, and 2 subgrid sizes) simulated activation maps were automatically generated and analyzed with the use of MatLab (Mathworks, Natick, MA, Potse et al., 2002).

## Results

### Average vector method applied to measured data

**Figure 3:**
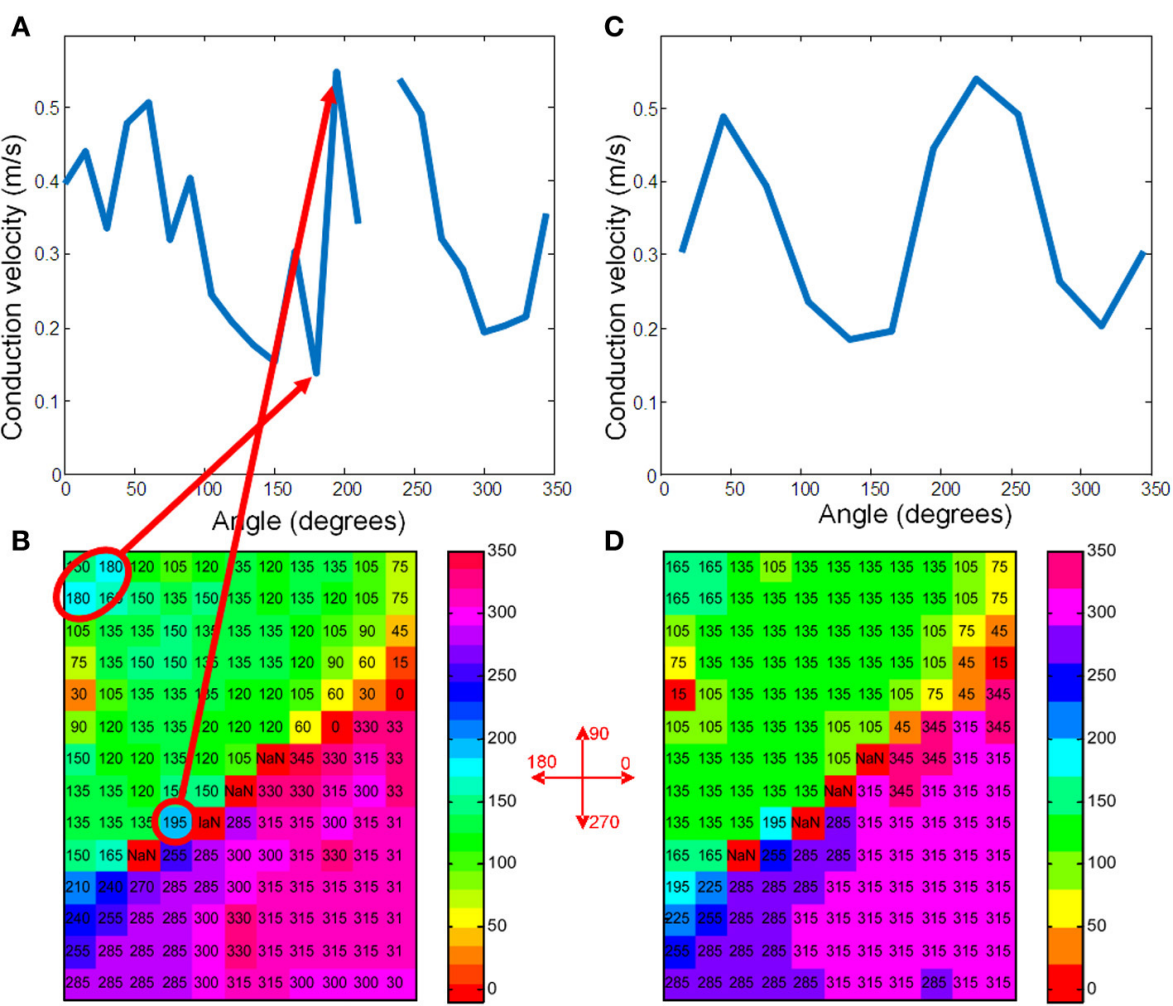
**(A)** Shows the average conduction velocities in the vector-bins for the map of Figure 2 when using 15° bins. **(C)** Shows the raw data with the vectors rounded to 15°. The grid positions that correspond to the highest and lowest velocities are marked with a red ellipse. Definition of the angles is indicated in between panels (**B**) and (**D**). **(B,D)** Give the same information for 30° bins. The two maxima of the sinusoidal curve correspond to the longitudinal velocity and the minima to the transversal velocity (Bayly et al., 1998).

For the average method to give reliable results the vectors need to be collected in bins that each contain enough vectors that have very similar directions. In Figures 3A–D the vector angles of the data from Figure 2 are binned (rounded) to 15 and 30°, respectively. The upper panels (a and b) show the average conduction velocity per bin, with a gap if there was no vector in a direction that corresponds to that bin. The numbers and colors in the lower panels represent the vector angle relative to the zero angle (“east,” as indicated by the cross between b and d). The red circles show the sites where the largest and smallest conduction velocities were recorded. With binning set at 15° (Figure 3A), the calculated maximum conduction velocity (about 0.55 m/s) is recorded at a site close to the pacing site, whereas the slowest conduction velocity (about 0.11 m/s) is recorded at a site near the margin of the grid. Figure 3B shows that using bins of 30°, the profile of conduction velocities is more smooth than in (Figure 3A) and now leads to a maximum CV of 0.48 m/s at 230° and a minimum CV of 0.2 m/s at 320°. If the single vector method is used the CV_l_ and CV_t_ are 0.47 and 0.19, respectively (see Figure 2C).

**Figure 4:**
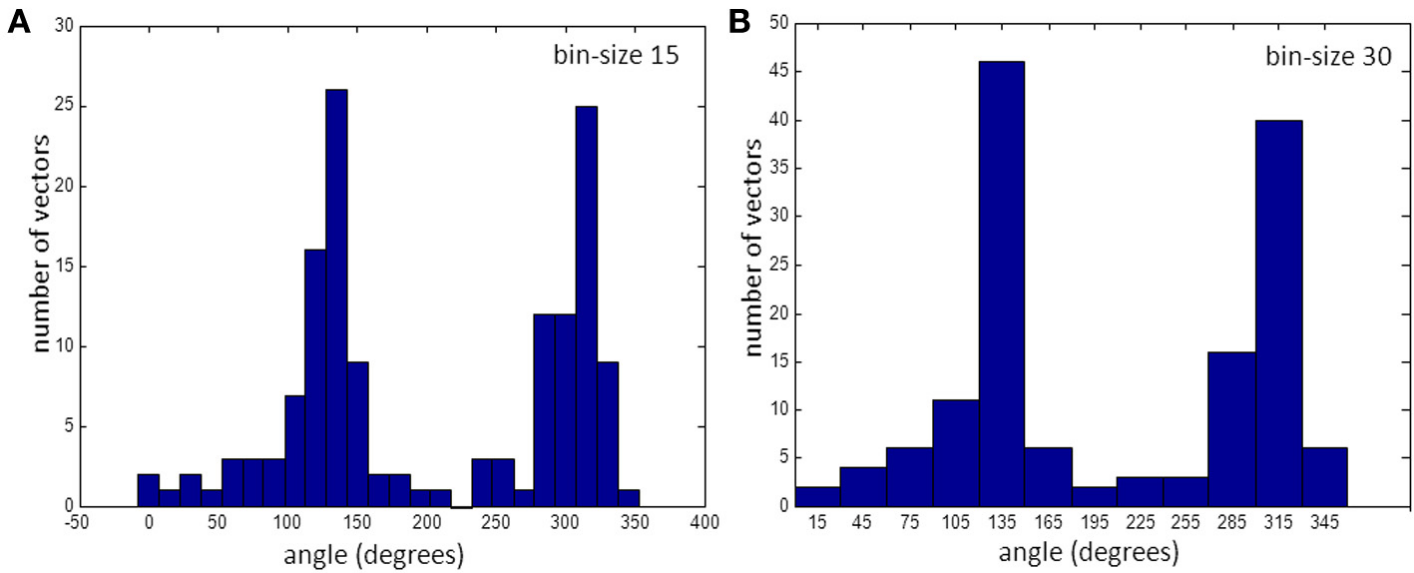
**Histogram of vectors in bins. (A)** for 15° bins and **(B)** for 30°. Note that the bin at 225° is empty. (See also gap in Figure 3A).

For this recorded map the number of observations in each bin varied from 0 to 27 when average vector directions were rounded to the nearest multiple of 15° (Figure 4A). There is one empty bin at an angle of 225°. Figure 4B shows that with a larger bin-size there are no empty bins and the number of vectors in every bin is at least 2.

### Simulations

**Figure 5:**
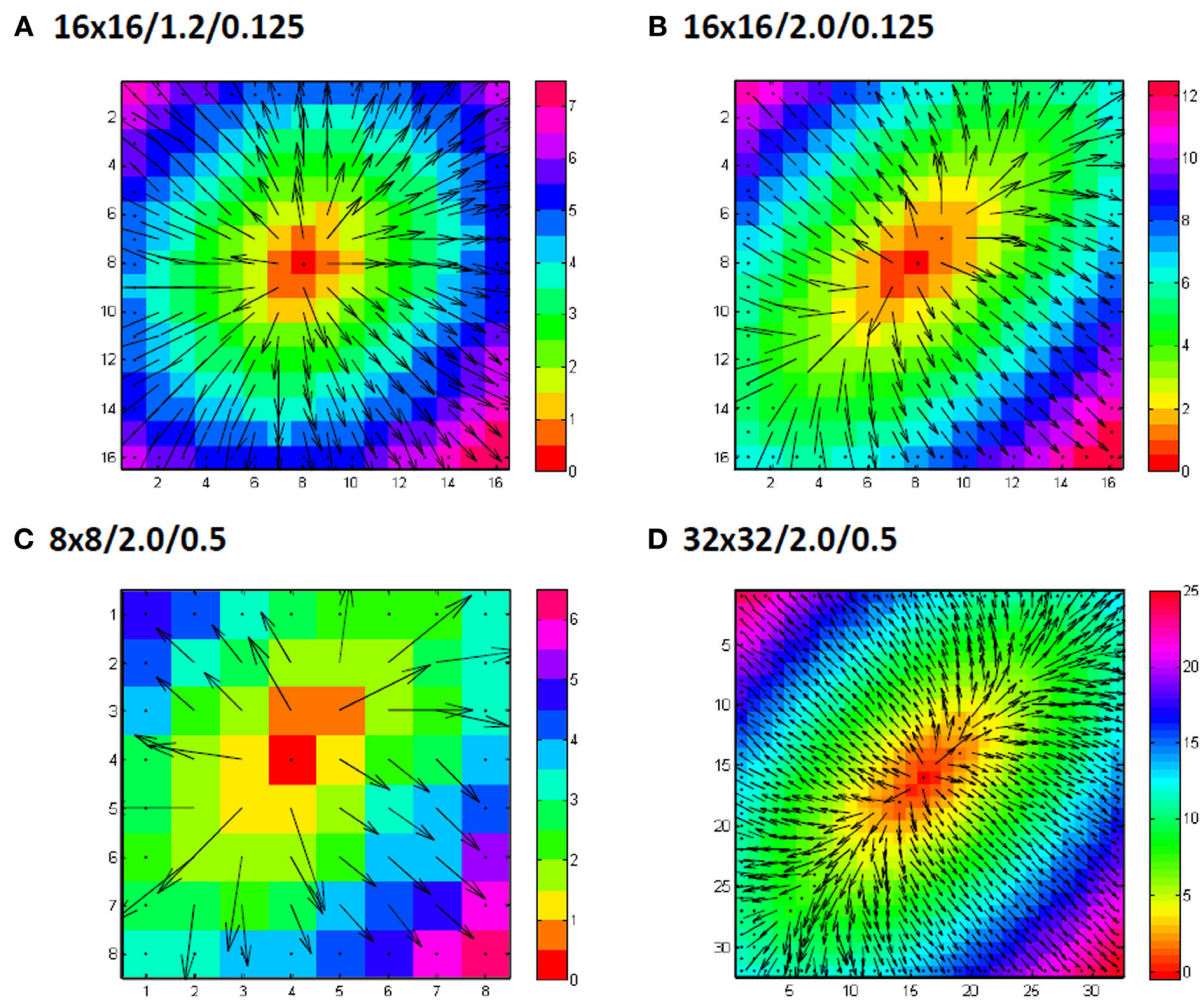
**Examples of grid sizes and anisotropic conduction velocity ratios**. Numbers indicate grid size/anisotropy ratio/added noiselevel in ms. In panel **(A)** an example of a 16 by 16 grid is shown for an anisotropy ratio of 1.2 (0.9 m/s longitudinal and 0.75 m/s transversal) with only 0.125 ms gausian noise added to the simulated activation times. Panel **(B)** shows another example, this time for a anisotropy ratio of 2 (0.9 m/s longitudinal, 0.45 m/s transversal). Panel **(C)** shows a simulation for a smaller grid (8 by 8) and panel **(D)** for a larger grid (32 by 32). In **(C)** and **(D)** also the simulated noise is larger with a standard deviation of 0.5 ms.

With the set of simulated map the effect of the various parameters on the accuracy of the calculated CV_l_ and CV_t_ was analyzed at two preselected bin-sizes of 15 and 30°. Figure 5 shows a couple of representative simulated activation vector maps.

**Figure 6:**
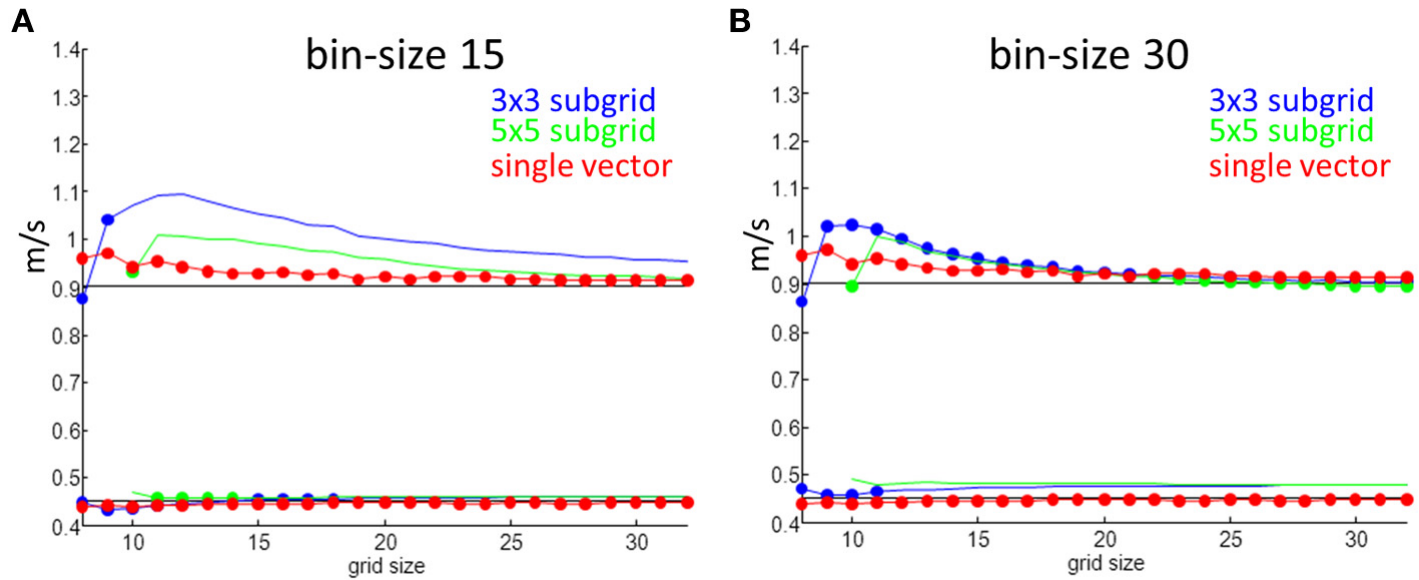
**Velocity measurements as a function of grid size**. Red are estimates from the single vector method, blue average method for a 3 by 3 subgrid and green for a 5 by 5 subgrid. Dots indicate that the true value is within one SD of the mean value. **(A)** Shows results for a bin size of 15° and **(B)** is for a bin size of 30°.

The simulation results for one set of values are shown in Figure 6. An anisotropic ratio of 2 and a noise level of 0.5 ms was used; Input CV_l_ and CV_t_ were set at 0.9 and 0.45 m/s, respectively, and activation times and maps were generated for each grid size ranging from 8 by 8 to 32 by 32. CV_l_ and CV_t_ were then calculated with the two methods. Compared to the single vector method, the “average vector” (blue and green lines) method has a larger overestimation of the longitudinal velocity for almost all grid sizes at a bin size of 15°, except for very large grids (approximately 22 by 22) and using 5 by 5 subgrids (Figure 5A). For grid-sizes smaller than about 16 by 16 for a bin-size of 30° (Figure 6B) values are also overestimated. The value used for the simulation is not within 1 SD from the mean for the average method for a bin size of 15, except for the smallest grid sizes where the SD is largest. The estimated values converge to the input value at grid sizes larger than 20, but at the same time the SD also becomes smaller and the true value does not come in range. The single vector method is closer to the actual value but has a larger SD than the 5 by 5 subgrid for larger grid sizes. Calculated CV_t_ is closer to the actual value than the CV_l_ for both methods but the SD for the average method is so small that there is consistent overestimation.

Overestimation in the longitudinal direction comes from measurement errors that result in one or two accidentally large vectors that are put in an (almost) empty bin. Overestimation in the transverse direction results from a large number of vectors that are a mix of transversal and longitudinal and therefore longer being put in the same bin as the pure transversal ones.

### Minimum size to prevent empty bins

At a bin-size of 15° and small grid-sizes it may happen that no vector is present in some of the bins (see Figure 4A), including those bins containing the angle representing fiber direction or, less likely, orthogonal to that. At large anisotropic ratios the chance of an empty bin in the direction of the fiber is larger than with a small anisotropic ratio. Thus, estimation of CV_l_ with the average vector method is more difficult and less reliable with smaller bin sizes, small grid sizes and larger anisotropy.

By varying the anisotropic ratio from 1.2 to 3 it was possible to estimate the minimum required size of a square grid that has a chance of less than 5% that the bin in the fiber direction contains no vector at all (1024 tests). The table shows the results of these simulations. The table shows for instance that a grid-size of at least 14 by 14 at an anisotropy-ratio of 2 and a noise-level of 0.5 ms is required to keep the chance below 5% that no vector is present in the bin representing the fiber direction (see Table 1). Required grid size increases with anisotropy. The addition of a realistic level of noise in the measurements again increases the minimum required size of the grid in order to prevent empty bins in the angle of the fiber direction.

**Table 1:** **Minimum required size of grid to have a 95% chance of having at least one vector in the bin representing the direction of the fibers**.

	**3 by 3**								**5 by 5**							
	**15° bins**				**30° bins**				**15° bins**				**30° bins**			
Anisotropy	1,2	1,5	2	3	1,2	1,5	2	3	1,2	1,5	2	3	1,2	1,5	2	3
Noise 1/8 ms	10	13	11	13	9	9	11	19	11	14	20	31	11	11	13	19
Noise 1/4 ms	10	13	13	15	9	9	12	19	12	14	20	25	11	11	13	19
Noise 1/2 ms	11	12	14	17	11	11	13	19	12	14	18	23	11	11	13	19
Noise 1 ms	12	13	15	19	11	11	13	17	12	14	17	23	11	11	13	17

*Numbers are for square grids, so “10” means at least a 10 by 10 grid*.

The single vector method is less sensitive to grid size, noise, and the degree of anisotropy. In all cases a mean closer than one SD from the actual value of CV_l_ and CV_t_ was computed.

## Discussion

### Possible problems with the average vector method applied to real data

If the “average vector” method is applied to real data, a number of problems may arise. The most important one in the example given is that neither the direction of the longitudinal (fastest) conduction nor the transversal conduction (slowest) conduction are along the long and short axis of the elliptical pattern. From Figure 3C it can be appreciated that the “longitudinal conduction velocity” (at 195°) is based on a single 3 × 3 subgrid observation that is very close to the site of stimulation. Close to the site of stimulation “latency” occurs which contorts activation pattern. Koller et al. (1995) on the other hand, the “transversal conduction velocity” is an average of two 3 × 3 subgrids in an area (see Figure 2) where isochronal lines are not equidistant. This area would have been excluded from analysis during single vector analysis. Another problem is that some bins are empty (Figure 3A). Unfortunately the bin most likely to be empty is the one in the direction of the longitudinal CV. Even if it is not empty, the number of local activation vectors is in general low, resulting is a large standard deviation. Contamination with vectors not purely in the direction of the fibers will result in a underestimation of the actual conduction velocity. In contrast, the transversal directions at 135 and 315° do contain a large number of vectors, but many of these are at sites at which activation is the result of a composite of transverse and longitudinal conduction. This results in a measurement with a small SD, but it may also suffer from an overestimation.

### Errors by non-elliptical spread of activations

The methods were compared during central stimulation under conditions in which it was assumed that no independent information on fiber direction is available. The single vector method is not applicable during other activation patterns, because the information on fiber direction cannot be derived from the activation map. Vector methods were originally developed to quantify the degree of heterogeneity in activation timing during anisotropic conduction (Lammers et al., 1990). Under those circumstances the average vector method may allow estimation of CV_l_ and CV_t_. One should, however, be aware that breakthrough patterns may yield unrealistic high CV_l_ values, because transmural rotation of myocardial fibers affects the epicardial activation pattern (Frazier et al., 1988; Schalij et al., 1992). Therefore, parts of the activation map where activation in deeper layers affects the layer where recordings are made, should not be used for analysis. In this simulation study a single layer with one global fiber orientation was assumed. For an automatic method based on the average vector method deviations from elliptical spread should also be detected automatically.

### Overestimates and underestimates of velocities using the average vector method

Both methods perform relatively well for the determination of transverse conduction velocity, and are not dependent on grid size, bin size (average vector method) and anisotropy. For small anisotropy and small grid sizes (<about 20 × 20) the average vector method underestimates transverse CV_t_ with more than 1 SD. This effect is larger for a bin size of 15 than for 30°. Longitudinal CV is often overestimated at a bin size of 15 by the average vector method when the grid size is low (<about 20 × 20). When the anisotropic ratio is large, the overestimation at smaller sizes is larger and also the chance that no vectors are in the fastest bin. Using a 5 by 5 grid in general improves the quality of the estimate, but larger grid sizes are required to be able to reliably measure at all. Using 30° bins instead of 15° gives better overall results.

## Limitations of this study

A limitation of this study is that these simulations assume that the activation spreads from a single stimulus point in the middle of the grid.

The assumption in this paper is that the myocardium is homogeneously anisotropic. As the example in Figure 2 shows, this is often not the case in real life. This is a minor problem for the “single vector” method, but for the “average vector” method it will result in a large number of vectors placed in the wrong bin. Worse, artifacts that result in overly long or small vectors in inhomogeneous parts can dominate the real vectors when the average is calculated from a small number of vectors. This will typically happen with the vectors in the direction of the fibers when the anisotropic ratio is 1.5 or more. The results obtained will therefore definitely be too large in case of the “average vector” method.

For the simulation experiments the direction of the longitudinal direction was known (i.e., 45°). Bin boundaries were selected in such a way that this 45° was in the middle of the bin. So, for 30° bins boundaries were 0, 30, 60,… and for the 15° bins −7.5, 7.5, 22.5, 37.5, 62.5,… For experiments in real hearts, this information is in general not known; using this knowledge may have positively skewed the results for the average method.

### Clinical applications of multielectrode mapping

Multielectrode mapping with regular grid electrodes in patients has been reported to determine the arrhythmogenic site of a tachycardia (Elvan et al., 2011) during open heart surgery or to assess the integrity of lines of conduction during minimal invasive procedures for AF (de Groot et al., 2012). Catheter approaches with 2D (Elvan et al., 2009) or 3D (Potse et al., 2004) multi electrode catheters have been reported as well, but in both these cases the inter-electrode distances are not constant, making the use of the average method difficult, if not impossible.

## Conclusions

This study compared two methods for the determination of conduction velocity.

The average vector method is a fast and easy tool to estimate the conduction velocities in myocardium. This method is often used in optical mapping experiments (Mironov et al., 2008) in which relatively large grid sizes are used (100 × 100). We demonstrate that under these conditions the method is highly reliable (after stimulation in the center of the grid), although a systematic underestimation of CV_l_ can be expected. When using small bin-sizes the average vector method will overestimate the longitudinal velocities. For larger grid-sizes it can overestimate the transversal velocities. Using a 5 by 5 subgrid requires larger grid sizes than 3 by 3, but the standard deviation of the results is smaller, and for smaller bin size the results are closer to the true value. Using larger subgrids does not fully solve the overestimation.

The single vector method performs better than the average vector method when grid size is small, bin size is small, sampling rate is low and anisotropy is large. This is at least in part related to the fact that (1) the identification of fiber direction allows selection of a single vector in the correct direction (and perpendicular to it); (2) the intrinsic assumption that CV_t_ is measured at right angles from CV_l_; and (3) that the largest possible distance over which linear conduction takes place is selected. The latter obviates the influence of breakthrough activation and allows greater time resolution (less influence of sampling rate). The single vector method is the preferred method when manual processing is an option.

### Conflict of interest statement

The authors declare that the research was conducted in the absence of any commercial or financial relationships that could be construed as a potential conflict of interest.
